# Photocatalytic Performance of Cu_x_O/TiO_2_ Deposited by HiPIMS on Polyester under Visible Light LEDs: Oxidants, Ions Effect, and Reactive Oxygen Species Investigation

**DOI:** 10.3390/ma12030412

**Published:** 2019-01-29

**Authors:** Hichem Zeghioud, Aymen Amine Assadi, Nabila Khellaf, Hayet Djelal, Abdeltif Amrane, Sami Rtimi

**Affiliations:** 1 Department of Process Engineering, Faculty of Engineering, Badji Mokhtar University, P.O. Box 12, 23000 Annaba, Algeria; hicheming@yahoo.fr; 2 Ecole Nationale Supérieure de Chimie de Rennes, Université de Rennes 1, CNRS, UMR 6226, Allée de Beaulieu, CS 50837, 35708 Rennes CEDEX 7, France; abdeltif.amrane@univ-rennes1.fr; 3 Laboratory of Organic Synthesis-Modeling and Optimization of Chemical Processes, Badji Mokhtar University, P.O. Box 12, 23000 Annaba, Algeria; khellafdaas@yahoo.fr; 4 Ecole des Métiers de l’Environnement, Campus de Ker Lann, 35170 Bruz, France; Hayet.Djelal@unilasalle.fr; 5 Ecole Polytechnique Fédérale de Lausanne, EPFL-STI-LTP, Station 12, CH-1015 Lausanne, Switzerland

**Keywords:** photocatalysis, reactive green 12, Cu_x_O/TiO_2_, polyester, HiPIMS, visible light LEDs

## Abstract

In the present study, we propose a new photocatalytic interface prepared by high-power impulse magnetron sputtering (HiPIMS), and investigated for the degradation of Reactive Green 12 (RG12) as target contaminant under visible light light-emitting diodes (LEDs) illumination. The Cu_x_O/TiO_2_ nanoparticulate photocatalyst was sequentially sputtered on polyester (PES). The photocatalyst formulation was optimized by investigating the effect of different parameters such as the sputtering time of Cu_x_O, the applied current, and the deposition mode (direct current magnetron sputtering, DCMS or HiPIMS). The results showed that the fastest RG12 degradation was obtained on Cu_x_O/TiO_2_ sample prepared at 40 A in HiPIMS mode. The better discoloration efficiency of 53.4% within 360 min was found in 4 mg/L of RG12 initial concentration and 0.05% Cu_wt_/PES_wt_ as determined by X-ray fluorescence. All the prepared samples contained a TiO_2_ under-layer with 0.02% Ti_wt_/PES_wt_. By transmission electron microscopy (TEM), both layers were seen uniformly distributed on the PES fibers. The effect of the surface area to volume (dye volume) ratio (SA/V) on the photocatalytic efficiency was also investigated for the discoloration of 4 mg/L RG12. The effect of the presence of different chemicals (scavengers, oxidant or mineral pollution or salts) in the photocatalytic medium was studied. The optimization of the amount of added hydrogen peroxide (H_2_O_2_) and potassium persulfate (K_2_S_2_O_8_) was also investigated in detail. Both, H_2_O_2_ and K_2_S_2_O_8_ drastically affected the discoloration efficiency up to 7 and 6 times in reaction rate constants, respectively. Nevertheless, the presence of Cu (metallic nanoparticles) and NaCl salt inhibited the reaction rate of RG12 discoloration by about 4 and 2 times, respectively. Moreover, the systematic study of reactive oxygen species’ (ROS) contribution was also explored with the help of iso-propanol, methanol, and potassium dichromate as ^•^OH radicals, holes (h^+^), and superoxide ion-scavengers, respectively. Scavenging results showed that O_2_^−^ played a primary role in RG12 removal; however, ^•^OH radicals’ and photo-generated holes’ (h^+^) contributions were minimal. The Cu_x_O/TiO_2_ photocatalyst was found to have a good reusability and stability up to 21 cycles. Ions’ release was quantified by means of inductively coupled plasma mass spectrometry (ICP-MS) showing low Cu-ions’ release.

## 1. Introduction

The textile industry is one of the largest consumers of water on our planet, and the second most polluting industry after the oil and gas industry [1,2]. Textile effluent containing synthetic dyes have toxic and carcinogenic compounds, which are stable and non-biodegradable due to the high molecular weight, the presence of azo bonds and amide groups in the molecular structure. Many of the chemical and physical treatment processes have shown insufficient results toward the degradation of theses pollutants. Recently, heterogeneous photocatalysis is one of the advanced oxidation processes that increasingly interests researchers. Photocatalysis was seen to transform/mineralize these synthetic dyes to lesser harmful by-products before their discharge into the environment. In the last decade, numerous works have been mostly focused on the designing and the development of new photocatalytic materials [3,4,5,6,7,8]. In this direction, different methods and preparations were investigated [3]. Sol–gel [4], hydrothermal [5], combined sol–gel/hydrothermal [6], chemical vapor deposition (CVD) [7], liquid phase deposition (LPD) methods [8] among many others were reported in the literature. In the aim of understanding the relationship between physico-chemical proprieties of photocatalyst and photocatalytic performances, various strategies have been adopted. Many studies investigated the effect of (1) semiconductor type such as TiO_2_, ZnO, and SnO_2_ [9,10]; (2) the number (mono-, bi-, or tri-doping) and the ratio of doping agents (N, P, Fe, Ag, etc.) [11,12,13,14]; (3) the support type such as polyester, cellulose, polypropylene, or polystyrene [15,16,17]; and (4) the substrate functional groups before the catalyst deposition [18,19]. It had been widely reported that band gap energy, surface area, particles size, and chemical stability were the most important parameters controlling the reactivity of a photocatalytic material [20,21,22]. TiO_2_ was reported to be the most suitable photocatalyst because of its high stability, low toxicity, low cost, chemical inertness, wide band gaps, and resistance to photo-corrosion [23,24,25]. Conventional direct current magnetron sputtering (DCMS) and high-power impulse magnetron sputtering (HiPIMS) have been used during the last decade to prepare photoactive thin films [26,27]. These films were reported to exhibit redox catalysis leading to bacterial inactivation [19,24,27], organic dyes degradation [25], antifungal [28], corrosion resistance [29], and self-cleaning [30]. Sequentially sputtered TiO_2_/Cu showed bacterial inactivation in the minute range [31,32]. Co-sputtered TiO_2_/Cu_x_O using HiPIMS was reported to lead to compact photoactive films showing reduced ions’ release [31]. The main goals of the present study are (i) to explore the HiPIMS deposition of Cu_x_O on TiO_2_ under-layer; (ii) to optimize the deposition parameters leading to stable thin film materials showing fast degradation of a toxic textile dye (Reactive Green 12) as target hazardous compound; (iii) to use the visible light light-emitting diodes (LEDs) system as efficient and economic light source; and (iv) to test the effect of the presence of some oxidant, mineral pollutant, or salts on the performance of the photoactive material. This latter goal is a step further to mimic real textile industry wastewater effluents normally presenting salts and oxidative agents. The photo-generation and the contribution of reactive oxygen species (ROS) are investigated in detail.

## 2. Experimental

### 2.1. Materials

Hydrogen peroxide (35 wt %, Merck KGaA, Darmstadt, Germany), potassium dichromate (>99 wt %, Carlo Erba Reagents S.A.S., Chaussée du Vexin, France), 2-propanol (>99.5%, Merck KGaA, Darmstadt, Germany), potassium peroxydisulfate (≥90 wt %, Merck KGaA, Darmstadt, Germany), chloroform (>99.97%, Acros-Organics, Thermo Fisher Scientific, Geel, Belgium), methanol (99.99%, Thermo Fisher Scientific, Geel, Belgium), copper (98.5 wt %, Merck KGaA, Darmstadt, Germany), and sodium chloride (99.5 wt %, Acros-Organics, Thermo Fisher Scientific, Geel, Belgium) were used. Reactive Green 12 dye (>99%, MW = 1837 g mol^−1^) procured from the textile manufacture of Constantine (Algeria) was used as received; the aqueous solutions were prepared using MilliQ water with a resistance of 15.0 MΩ·cm. The chemical structure and properties of Reactive Green 12 (RG12) dye were recently reported [18].

### 2.2. Catalyst Preparation

The Cu_x_O/TiO_2_ coatings were sputtered using reactive HiPIMS process. Ti and Cu targets were purchased from Lesker (Kurt J. Lesker Company Ltd., East Sussex, UK) (99.99% pure). The sputtering chamber was operated at a high vacuum with a residual pressure less than 4 × 10^−5^ Pa. This chamber was equipped with two confocal targets 5 cm in diameter. A HiP3 5KW Solvix generator (Advanced Energy, Fort Collins, CO, USA) was used for the HiPIMS deposition and was operated at an average power of 100 W (5 W·cm^−2^) with a pulse-width of 50 µs and a frequency of 1000 Hz. A TiO_2_ under-layer was sputtered (for 1 min) before the deposition of Cu_x_O to reduce the voids of polyester and to permit a high dispersion of Cu species on the surface [33]. The target-to-substrate distance was fixed at 10 cm in order to obtain homogeneous and adhesive Cu_x_O films. The sample holder was rotating at a speed of 18 rpm.

**Figure 1:**
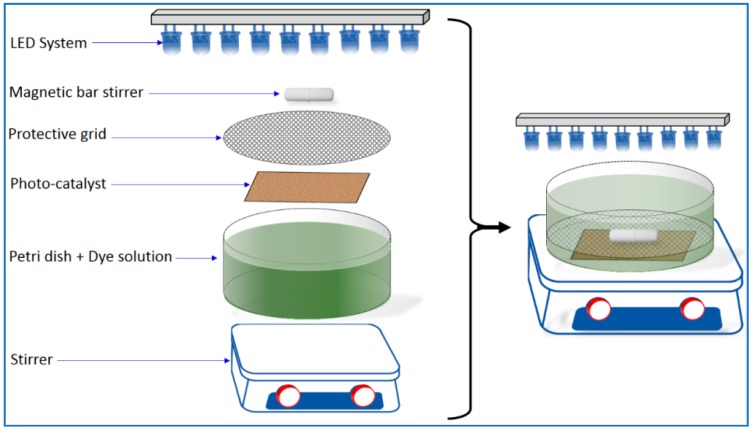
Photocatalytic apparatus for Reactive Green 12 (RG12) degradation.

Table 1 shows a summary about the prepared catalysts used in this study. Different current intensities were applied to the Cu-sputtering target—(1) HiPIMS mode (20 A, 40 A and 80 A) and (2) direct current mode (direct current magnetron sputtering, DCMS) at 300 mA. The Cu-deposition times were 5, 10, 20, and 100 s. The obtained photocatalytic thin layers were used in the degradation of 4 mg/L of RG12 solution, as shown in Figure 1.

### 2.3. Characterization of Materials

The UV–Vis spectra of all samples were recorded using a Varian Cary^®^50 UV–Vis spectrophotometer (Agilent, Les Ulis, France), where the spectra were obtained at the wavelength range of 200–800 nm (λ_max_ = 615 nm).

Transmission electron microscopy (TEM) of the sputtered polyester (PES) fabrics was carried out using a FEI Tecnai Osiris (Thermo Fisher Scientific, Hillsboro, OR, USA) operated at 200 kV. The spot size was set to 5 µm, dwell time 50 µs, and the real time of 600 s. The samples were embedded in epoxy resin (Embed 812) and cross-sectioned with an ultra-microtome (Ultracut E) up to thin layers of 80–100 nm thick. These thin layers were then placed on TEM holey carbon grid in order to image the samples.

X-ray diffraction (XRD) INEL EQUINOX instrument (INEL, Stratham, NH, USA), power 3.5 kW and coupled with a CPS120detector (INEL, Stratham, NH, USA) was used to register peaks from 2 to 120 θ.

### 2.4. Photocatalytic Experiments

The photocatalytic activity of the synthesized photocatalyst was evaluated by following the degradation of 15 mL of the RG12 dye solution in petri dish as a reactor under magnetic stirring (see Figure 1). The reaction system included an ultraviolet light-emitting diodes (LEDs) system (visible light LEDs, Innolux, Santa Clara, CA, USA) as an irradiation source (λ = 420 nm) with a measured intensity of 1 mW/cm^2^. The initial RG12 solution was stirred in the dark for 30 min to reach adsorption–desorption equilibrium before the LEDs irradiation was ON. The concentration of dye solution samples (V = 3 mL) was analyzed using a UV–Vis spectrophotometer (Agilent, Les Ulis, France) at regular time intervals. The protective grid is made of stainless steel and is used to protect the catalyst from the possible mechanical damage caused by the stirrer.

The RG12 discoloration efficiency (%) of the material was evaluated by the following Equation (1)

(1) $$n(\%)=(\frac{A_{0}-A_{t}}{A_{0}})\times 100$$

where *A*_0_ and *A_t_* are the initial intensity of absorbance peak at λ_max_ (λ_max_ = 615 nm) and the intensity of absorbance peak at time *t* in UV–Vis spectra of RG12, respectively.

The coated fabrics were also tested for stability by testing their recycling performance. The ions released from the fabrics were quantified using inductively coupled plasma mass spectrometry (ICP-MS) using Finnigan^™^, Element2 high-performance high-resolution ICPMS model (Zug, Switzerland). The ICP-MS resolution was of 1.2 × 10^5^ cps/ppb with a detection limit of 0.2 ng/L. Clean Teflon bottles were used to prepare the calibration standards through successive dilutions of 0.1 g L^−1^ ICPMS stock solutions (TechLab, Metz, France). The washing solution from the samples were digested with nitric acid 69% (1:1 HNO_3_ + H_2_O) to remove the organics and to guarantee that there were no remaining ions adhered to the reactor wall.

## 3. Results and Discussion

### 3.1. Effect of the Photocatalyst Preparation Parameters on the RG12 Discoloration and the Microstructure

**Figure 2:**
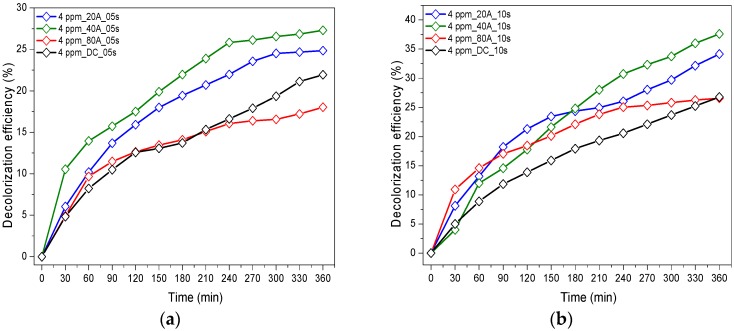

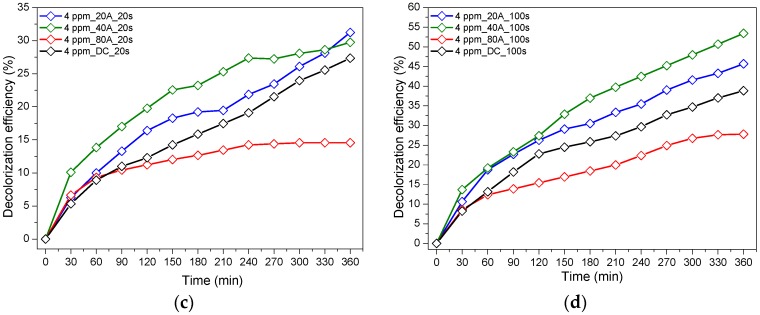
Photocatalytic degradation of RG12 with different current intensity used in photocatalyst preparation (initial pollutant concentration 4 mg/L) on (**a**) samples sputtered for 5 s on polyester (PES); (**b**) samples sputtered for 10 s; (**c**) samples sputtered for 20 s; and (**d**) samples sputtered for 100 s.

The photocatalytic degradation of RG12 under the LEDs is shown in Figure 2. We noted that when applying a current intensity of 40 A to the target, the resulting thin film showed the fastest RG12 degradation followed by HiPIMS at 20 A, DCMS (300 mA), and HiPIMS at 80 A with 100 s as deposit time.

From another hand, at this value of current intensity (40 A), increasing the sputtering time led to an increase in the photocatalytic discoloration efficiency of RG12, where 27.3%, 33.1%, 37.6%, and 53.4% dye elimination after 360 min under irradiation was observed with 05, 10, 20, and 100 s deposition time, respectively, as shown below in Figure 2. The TiO_2_ under-layer did not show any photocatalytic activity (3–6% RG12 removal). This is in accordance with previous results found for methylene blue [25]. This can be attributed to the low amount of TiO_2_ (and active sites) available for RG12.

**Figure 3:**
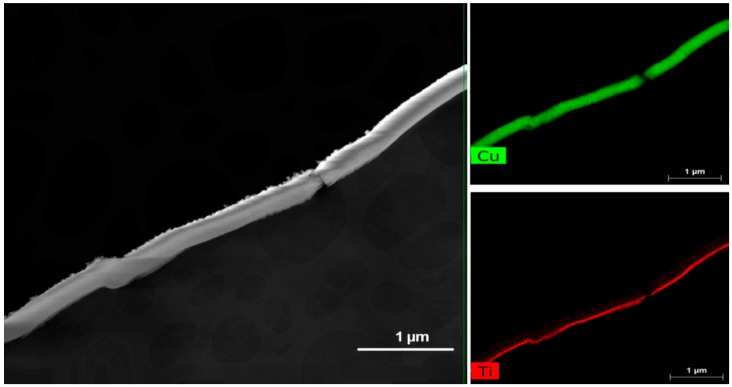
TEM imaging of Cu_x_O/TiO_2_ deposited by high-power impulse magnetron sputtering (HiPIMS) at 40 A.

This can be attributed to the optimal mass-to-volume ratio of the HiPIMS deposited film at 40 A. It has been reported that small-sized nanoparticles induce favorable photocatalytic bacterial inactivation kinetics due to the large surface area per unit mass [24]. The distribution of Cu_x_O nanoparticles on the TiO_2_ under-layer on the polyester was found to be uniform and did not induce cracks on the substrate. Figure 3 shows the TEM imaging of the sputtered layers (HiPIMS at 40 A) on polyester (PES). The TiO_2_ under was sputtered to reduce the porosity of the PES leading to the continuous distribution of the Cu_x_O upper layer [31,33].

The charge transfer between the TiO_2_ and the Cu_x_O at the surface and the organic pollutant depends on the diffusion length of the photo-generated charges at the interface of the film under light irradiation. The charge transfer/diffusion is also a function of the TiO_2_ and of the Cu-particles’ size and shape as previously reported [34,35].

X-ray diffraction of the sputtered Cu_x_O/TiO_2_ on PES (sputtered for 100 s at 40 A) showed the absence of sharp peaks that can be attributed to the Cu_x_O clusters. This reflected the low crystallinity of the deposited Cu clusters. The absence of clear crystal phase could be attributed to the short sputtering time leading to the formation of very small Cu nanoparticles/clusters. The low Cu and Ti loadings led to ~80 nm thin film on the porous PES. The diffractogram showed a high noise-to-signal ratio due to the low amount of Cu species on the PES.

### 3.2. Effect of key Parameters: Pollutant Concentration and Surface-Area to Volume Ratio

**Figure 4:**
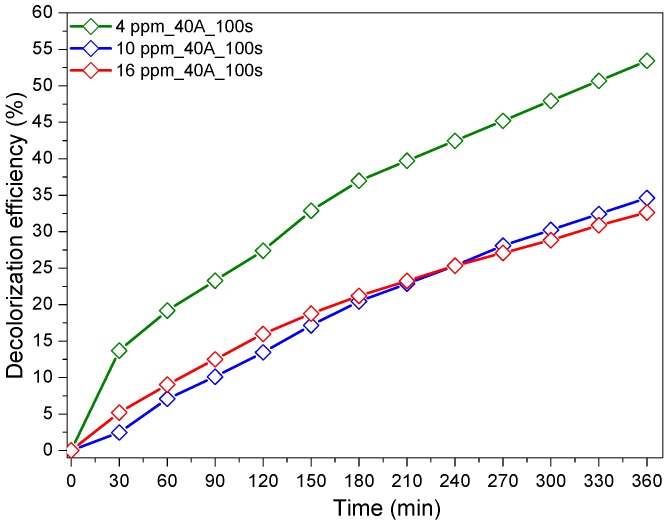
Photocatalytic degradation of RG12 with different initial concentrations (current intensity, 40 A and sputtering time, 100 s).

Figure 4 shows the photocatalytic RG12 discoloration when using different initial dye concentrations. It is readily seen from Figure 4 that the discoloration is faster for lower initial concentrations, with optimal kinetics for 4 mg/L. This can be explained by the unavailability of active site for the adsorption of all dye molecules and their degradation at high initial concentrations [25]. Furthermore, the increase in dye concentration led to an increase in medium opacity and then less permeability to the applied light, which reduced the photo-generated reactive species important for the photocatalytic process [25,36]. It has been recently reported that porous Cu_2_O nanostructures were also able to adsorb ions such as radioactive iodine issued from nuclear fission [37].

**Figure 5:**
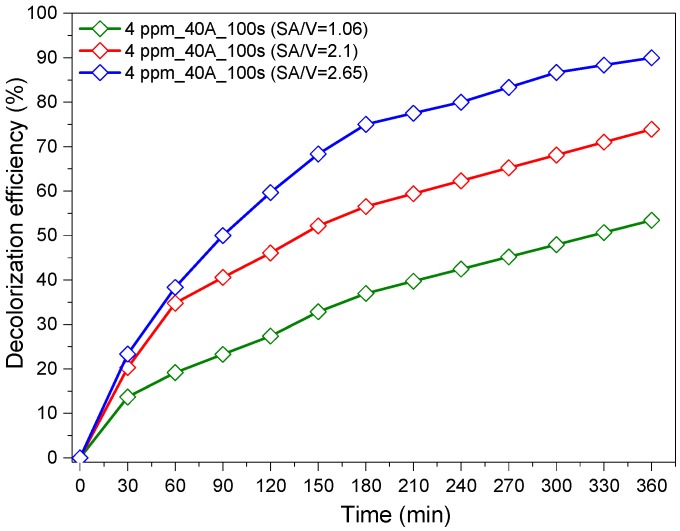
Photocatalytic degradation of RG12 with different catalyst surface area to volume ratio (initial concentrations, 4 mg/L; current intensity, 40 A; and sputtering time, 100 s).

In order to investigate the effect of the photocatalyst surface (in other words, the available catalytic active sites), the effect of increasing surface area to volume (dye volume) ratio (SA/V) on the photocatalytic efficiency was studied using higher ratios for the discoloration of 4 mg/L RG12. We noted that the used photocatalytic surface in the previous experiment was fixed to 16 cm^2^, which gave a surface area to volume ratio (SA/V) of 1.06 cm^−1^. The results are shown in Figure 5. It was readily seen that increasing SA/V increased the discoloration efficiency. This can be attributed to the increase in the number of active sites available for RG12 molecules and intermediate by-products’ adsorption and degradation. A ratio of 1.06 showed 53.4% of discoloration efficiency; however, a ratio of 2.66 cm^−1^ led to 90.1% under visible light LEDs irradiation for 360 min. Similar results were reported by Huang et al. for the methyl orange discoloration on Pt-modified TiO_2_ supported on natural zeolites [38].

### 3.3. Implication of Radicals and ROS Species within RG12 Discoloration

The contribution of individual reactive oxidizing species (^•^OH, O_2_^•–^, and h^+^) in the RG12 degradation was studied using selective scavengers (MeOH and iso-propanol for ^•^OH, potassium dichromate for O_2_^•−^, and CHCl_3_ and MeOH for electronic holes). The choice of selective scavenger was based on the second-order kinetic rate constant of the reaction between the ROS and each scavenger as shown in Supplementary Materials Table S1.

**Figure 6:**
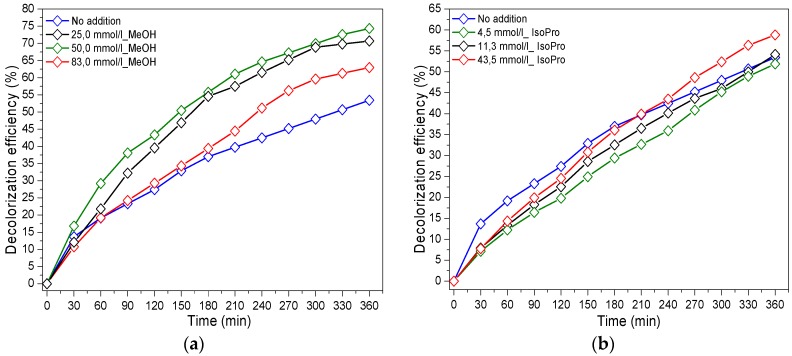

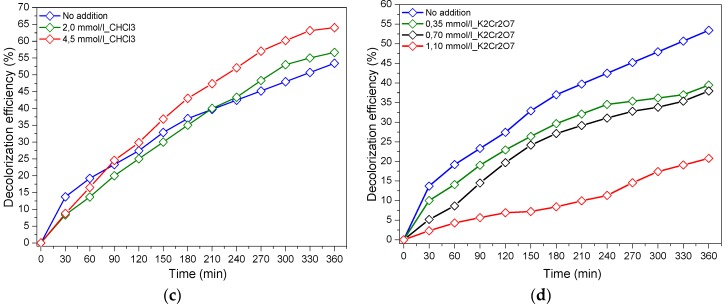
Photocatalytic degradation of RG12 in the presence of (**a**) methanol; (**b**) iso-propanol; (**c**) chloroform; and (**d**) potassium dichromate. (Initial RG12 concentration = 4 mg/L; sample, HiPIMS at 40 A; and sputtering time, 100 s.)

Figure 6a shows that the presence of MeOH (holes scavenger) in reaction medium enhanced the discoloration efficiency of the reactive dye. Furthermore, increasing the MeOH amount led to the acceleration of the photocatalytic RG12 abatement until an optimum value of alcohol of 50 mmol/L leading to 74.3% of discoloration efficiency after 360 min of irradiation.

On the other hand, as shown in Figure 6b, the presence of iso-propanol in the reaction medium slightly enhanced the photocatalytic RG12 discoloration, where 58.8% of RG12 was destroyed with 43.5 mmol/L of alcohol after 360 min compared to 53.4% without scavenger addition.

Figure 6a,b shows that the presence of alcohol (MeOH or iso-propanol) slightly enhanced the photocatalytic reaction rate, which can be explained by the inhibitory effect played by hydroxyl radicals in the degradation mechanism of RG12. Similar results were found by Rong and Sun in their work reported on the photocatalytic degradation of triallyl isocyanurate (TAIC) [39]. The additional effect in the inhibition of discoloration efficiency in the presence of MeOH compared to iso-propanol can be attributed to the holes’ effect because of the high reactivity of iso-propanol with the hydroxyl radicals (k_iso-prop,_^•^_OH_ = 1.9 × 10^9^ M^−1^ S^−1^) [40] compared to the MeOH (k_MeOH,_^•^_OH_ = 9.7 × 10^8^ M^−1^ S^−1^) [41].

It is worth to mention at this level that Di Valentin and Fittipaldi [42] studied the photo-generated hole scavenging using different organic adsorbates on TiO_2_. They established a scale of scavenging power showing glycerol > tert-butanol > iso-propanol > methanol > formic acid. This suggested that methanol could exhibit electronic-holes scavenging, but negligible compared to the ^•^OH-radical scavenging. Furthermore, Shen and Henderson [43] reported on the molecular and dissociative forms of methanol on TiO_2_ surface for holes scavenging. They showed that methoxy (dissociative form of methanol) effectively scavenged the photo-generated holes, and not methanol itself. This did not allow an unequivocal separation between ^•^OH-radical and h^+^ scavenging when using methanol.

Figure 6c shows that the presence of 4.5 mmol/L of chloroform slightly improved the discoloration efficiency of RG12 by 11% after 360 min under light. This enhancement can be attributed to the holes-scavenging characters of Cl ions generated from the chloroform decomposition/dissolution.

Oxygen superoxide anion (O_2_^•−^) is one of the very important radicals studied because of its crucial role in the photocatalytic process. The contribution of this photo-generated radical was studied by adding potassium dichromate (K_2_Cr_2_O_7_) to the reaction medium. The results of adding are shown in Figure 6d. The presence of potassium dichromate decreased the reaction rate of RG12 discoloration, where a reduction of 32.7% in discoloration efficiency was recorded in the presence of 1.1 mmol/L K_2_Cr_2_O_7_. An inversely proportional relationship between the added quantity of K_2_Cr_2_O_7_ and the discoloration efficiency of RG12 was also noticed. This can be explained by the positive role played by O_2_^•−^ in the elimination of RG12 textile dye.

### 3.4. Effect of the Addition of Oxidizing Agents: Cases of H_2_O_2_ and K_2_S_2_O_8_

**Figure 7:**
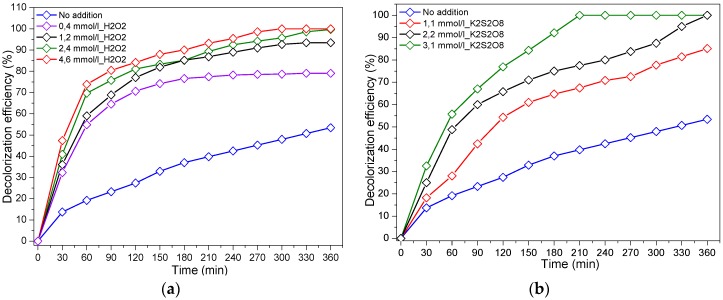
Photocatalytic degradation of RG12 in the presence of (**a**) hydrogen peroxide (H_2_O_2_) and (**b**) potassium peroxydisulfate (K_2_S_2_O_8_). (Initial RG12 concentration = 4 mg/L; sample, HiPIMS at 40 A; and sputtering time, 100 s.)

In order to enhance the photocatalytic reaction, several intensification ways can be tested. The effect of the adding oxidants such as hydrogen peroxide (H_2_O_2_) and potassium peroxydisulfate (K_2_S_2_O_8_) in the reaction medium (mimicking real textile industry effluents) on the photocatalytic reaction kinetics was investigated. Figure 7a shows that adding hydrogen peroxide (H_2_O_2_) strongly improved the reaction rate, where 47.4% and 100% discoloration were, respectively, achieved after 30 and 300 min in the presence of 4.6 mmol/L H_2_O_2_. However, only 48% of discoloration efficiency was noticed without any addition after 300 min under light. The strong enhancement caused by the presence of H_2_O_2_ can be explained by (i) the participation of H_2_O_2_ in the generation of ^•^OH from one hand [44] and (ii) the reduction of the recombination rate as shown below in Equations (2)–(4) from another hand [45,46]. It is worth to mention at this level that the ^•^OH is 1.5 times more oxidant than H_2_O_2_ and 1.4 times more than ozone [47,48].

(2) $$e^{-}{}_{cb}+H_{2}O_{2}\to 2HO^{•}$$

(3) $$O_{2}{{}^{•}}^{-}+H_{2}O_{2}\to {}^{•}OH+OH^{-}+O_{2}$$

(4) $$hv+H_{2}O_{2}\to 2{(}^{•}OH)$$

The effect of persulfate ions on the photocatalytic degradation of RG12 was investigated by varying its concentration from 1.1 to 3.1 mmol/L. Figure 7b shows that the presence of potassium peroxydisulfate (K_2_S_2_O_8_) drastically increased the reaction rate of RG12 discoloration, where 67.5% and a full discoloration (100%) were achieved within 210 min with 1.1 and 3.1 mmol/L S_2_O_8_^2−^, respectively. This can be due to the electron-scavenging properties of S_2_O_8_^−^ [49], which led to the formation of sulfate radical anions (SO_4_^•−^) with a high oxidizing power (*E*° = 2.6 eV) by S_2_O_8_^2−^ reduction according to the Equations (5) and (6) [50].

(5) $$S_{2}O_{8}{}^{2-}+e^{-}{}_{cb}\to SO_{4}{}^{•-}+SO_{4}{}^{2-}$$

(6) $$SO_{4}{}^{•-}\to SO_{4}{}^{2-}+h_{vb}{}^{+}$$

The effect of SO_4_^•−^ in the enhancement of RG12 discoloration can be rationalized in (i) the prevention of electron/hole recombination rate resulted from the interaction with conduction band electrons leaving behind positives holes [51]; (ii) the abstraction of a hydrogen atom from saturated carbon [44]; (iii) the generation of hydroxyl radicals by interaction with H_2_O molecules according to Equation (7) [52]; and (iv) the possible reaction of sulfate radical anions and the dye molecules by direct attack [53].

(7) $$SO_{4}{}^{•-}+H_{2}O\to SO_{4}{}^{2-}+{}^{•}OH+H^{+}$$

At this stage, it is worth to mention that copper oxide can be easily “sulfated”. This may affect the catalytic performance of the catalyst.

### 3.5. Effect of Different Water Matrices on RG12 Degradation

**Figure 8:**
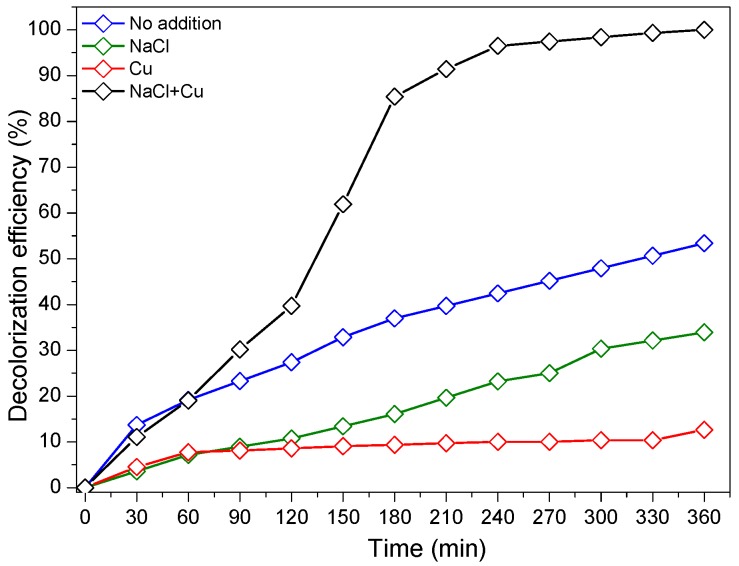
Photocatalytic degradation of RG12 in the presence NaCl and Cu. (Initial RG12 concentration = 4 mg/L; sample, HiPIMS at 40 A; and sputtering time, 100 s).

In order to approach a real case of textile effluent, the effect of mineral ions’ and salts’ presence on the photocatalytic reaction rate was investigated by testing both Cu and NaCl as inorganic ions. Cu and NaCl were tested separately, and then combined together. It is readily seen from Figure 8 that the presence of Cu has a negative effect on the discoloration efficiency which can be explained as follows: (i) the possible complexation of Cu and organic species or some intermediate by-products [54]; (ii) according to the work of Kumawar et al. [55], there is an optimum concentration when adsorbed metal ions change the behavior from enhancer to inhibitor because of changing surface charge of the photocatalyst or the target pollutant. Similar results on Cu-inhibitory effect was found by Tercero Espinoza et al. [56]; and (iii) the presence of transition metal ions can alter the electron-transfer pathway, decrease the reduction of oxygen, and even suppress the degradation [57].

The addition of NaCl as chloride ions source (Cl^−^) to the dye solution in our experiment led to a reduction of discoloration efficiency by about 20% after 360 min under LEDs light (420 nm).

The effect of anions in the photocatalytic medium has been reported to have an inhibitory characters due to (1) the competitive effect between the anions and the target pollutant toward the active sites available on the surface of the photocatalyst [58]; (2) the anion radicals’ scavenging properties such as holes and hydroxyl radicals, which led to the formation of ionic radicals such as Cl^•^, NO_3_^•^, and HCO_3_^•−^ less reactive than ^•^OH [20]; and (3) the h^+^-scavenging proprieties of chloride ions according to Equation (8) [59].

(8) $$Cl^{-}+h^{+}{}_{vb}\to Cl^{•-}+H^{+}$$

The simultaneous addition of Cu and NaCl (both at the same time) led to a drastic enhancement in the discoloration rate of RG12, where 98% of RG12 discoloration was observed within 360 min of illumination, as shown in Figure 8.

RG12 removal can be influenced by the multitude of species present in the real wastewater effluent. Mathematical simulation of the degradation of RG12 was seen to fit a second-order model as described by the equation (see supplementary Table S1):

$$\frac{d[\mathrm{RG}12]}{dt}=-k\left[\mathrm{RG}12\right]\left[\mathrm{AS}\right]$$

where [AS] is the steady-state concentration of actives species (radical hydroxyl, sulfate ion radical, reactive oxygen anion superoxide, etc.), [RG12] is the concentration of RG12 in water, *k* is the second-order rate constant and *t* is the reaction time.

Taking into account the instantaneous concentrations of the photo-generated ROS, the kinetics of the degradation of RG12 in water can be described according to the pseudo-first-order equation as given below:

$$\left[\mathrm{RG}12\right]\left(t\right)={\left[\mathrm{RG}12\right]}_{0}\exp^{-k_{app}.t}$$

where *k_app_*, is the pseudo-first-order apparent rate constant (min^−1^) and it was obtained by linear regression of ln(*C_t_/C_o_*) vs. time *t*.

**Figure 9:**
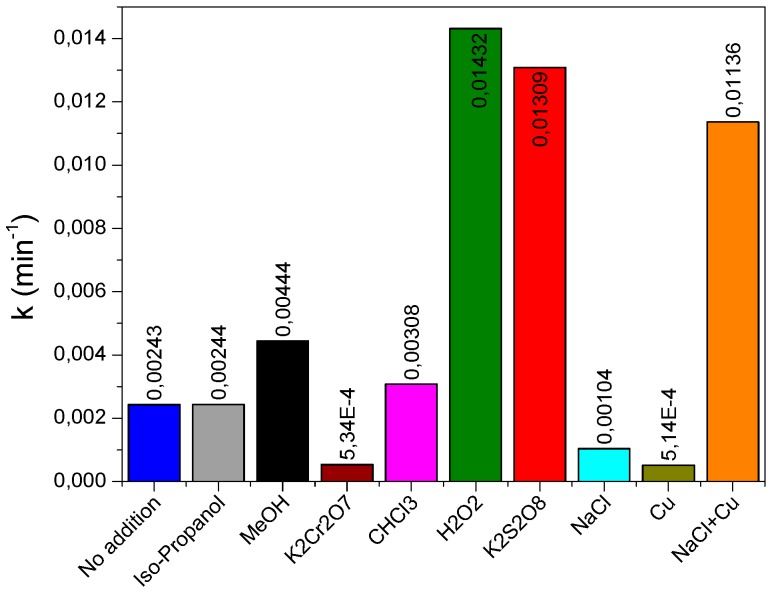
Reaction rate constant of RG12 photocatalytic degradation in the presence of different chemical products.

Figure 9 summarizes the reaction rate constants *k_app_* of RG12 degradation with and without the addition of various chemicals (scavengers, oxidants, or inorganic pollutants). The constants varied from very low values of 0.00051 min^−1^ in the presence of Cu ions to very high values (0.0143 min^−1^) in the presence of H_2_O_2_. In the presence of H_2_O_2_, there was an enhancement of 7 times of the RG12 discoloration rate followed by a 6 times’ enhancement with K_2_S_2_O_8_. It is readily seen that the constant rate of reaction in the presence of methanol (0.004 min^−1^) is higher than with the addition of iso-propanol (0.002 min^−1^).

**Figure 10:**
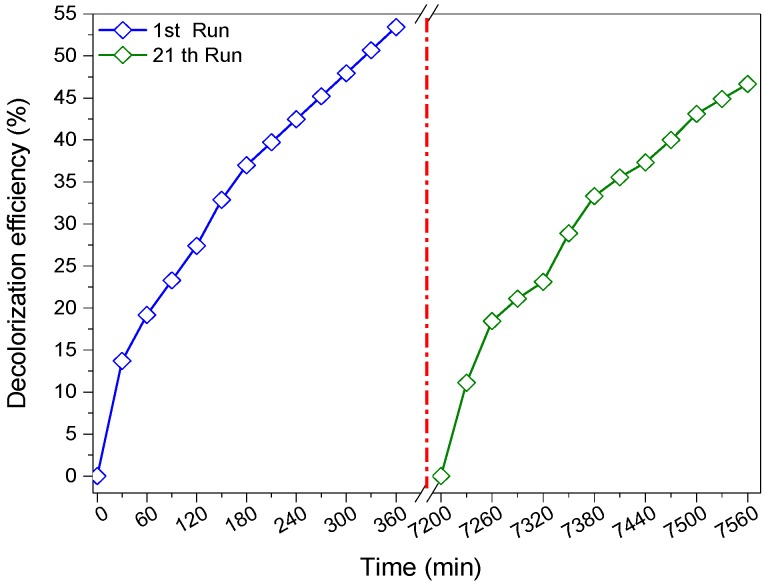
Recycling runs of the catalyst for the photocatalytic degradation of RG12 up to 21 runs (initial concentrations: 4 mg/L; catalyst dose, 1 sheet; current intensity, 40 A; and sputtering time, 100 s).

In the aim to evaluate the stability and the reusability of the synthesized material supported on PES used in this study, successive photocatalytic cycles were applied for the degradation of RG12 (4 mg/L in each experiment) with the same catalyst. As it can be seen in Figure 10, the photocatalyst has a good stability under LEDs illumination approved by the slight decrease in discoloration efficiency (about 7%) after 21 runs.

Table 2 shows the released Cu and Ti ions from the fabrics after 1, 5, 10, and 21 washing cycles. The results presented in Table 2 are cumulative quantification of the released ions during all the cycles. It is readily seen from Table 2 that the Ti ions are found at very low quantities during the first 5 cycles and then reduced drastically to almost zero ppb at the end of the recycling experiment. Table 2 also shows that the Cu ions’ release slows down at the end of the recycling (after the 10th cycle) to stabilize at about 2–3 ppb/cycle. These results were slightly similar to previous reports using HiPIMS deposition of Cu_x_O or TiO_2_/Cu deposited on polyester for photocatalytic applications [31,15,60]. The cumulative ions’ release is still far below the threshold fixed by regulatory bodies for Cu and/or Ti ions.

## 4. Conclusions

Cu_x_O thin films deposited using HiPIMS on polyester under different sputtering energies were successfully synthesized. The sputtering mode and the applied current intensity were optimized. The photocatalytic performance of the photocatalyst was evaluated for the degradation of RG12 under visible light LEDs irradiation with different initial dye concentrations and surface area to volume ratios. The effect of different concentrations of various chemicals (scavengers, oxidants, or inorganic ions) on the photocatalytic process was also studied mimicking a real textile effluent containing a large variety of compounds. The monitoring of ROS showed that the superoxide ions played the main role in RG12 degradation contrary to hydroxyl radicals and the photo-generated holes. The presence of H_2_O_2_ or K_2_S_2_O_8_ presented a high oxidizing power, enhancing the photocatalytic activity of the sputtered catalyst. The presence of Cu as mineral pollution and NaCl as salt decreased the reaction rate, but the addition of both of them (Cu and NaCl salts) improved the removal efficiency of the studied pollutant. The recycling of the catalyst showed a high stability of the catalyst up to 21 RG12 discoloration cycles. ICP-MS showed stable ions’ release after the 5th cycle for both ions. This allows potential industrial applications of the reported HiPIMS coatings in the future.

## Figures and Tables

**Table 1:** Summary of the prepared catalysts used in this study.

Catalyst	Sputtering Mode	Sputtering Time of Copper Upper Layer (s) *	Sputtering Power
Cu_x_O/TiO_2_	DCMS/DCMS	5, 10, 20, and 100	0.5 A/0.3 A
Cu_x_O/TiO_2_	HiPIMS/DCMS	5, 10, 20, and 100	20 A/0.3 A
Cu_x_O/TiO_2_	HiPIMS/DCMS	5, 10, 20, and 100	40 A/0.3 A
Cu_x_O/TiO_2_	HiPIMS/DCMS	5, 10, 20, and 100	80 A/0.3 A

* The TiO_2_ under-layer was sputtered for 1 min.

**Table 2:** Inductively coupled plasma mass spectrometry (ICP-MS) quantification of ions’ release during the recycling experiment.

	Cu Ions	Ti Ions
Cycle 1	17	6
Cycle 5	29	9
Cycle 10	32	10
Cycle 21	34	10

## Supplementary Materials

The following are available online at http://www.mdpi.com/1996-1944/12/3/412/s1, Table S1: Second order rate constants of different scavengers with radical species.
