# Supplementary material for: Photocatalytic Performance of CuxO/TiO2 Deposited by HiPIMS on Polyester under Visible Light LEDs: Oxidants, Ions Effect, and Reactive Oxygen Species Investigation
Source: Materials (Basel). 2019 Jan 29;12(3):412. doi: 10.3390/ma12030412 (PMC6385099; doi:10.3390/ma12030412)
Supplement: Supplementary file 1 [file materials-12-00412-s001.pdf]

Supplementary materials for:

Photocatalytic performance of Cu_xO/TiO_2 deposited by HiPIMS on Polyester under visible light LEDs: oxidants, ions effect and reactive oxygen species investigation

Hichem Zeghioud[1], Aymen Amine Assadi[2*], Nabila Khellaf[3], Hayet Djelal[4], Abdeltif Amrane[3] and Sami Rtimi[5**]

[1] Department of Process Engineering, Faculty of Engineering, Badji Mokhtar University, P.O. Box 12, 23000 Annaba, Algeria
[2] Université de Rennes 1, ENSCR, CNRS, UMR 6226, Allée de Beaulieu, CS 50837, 35708 Rennes Cedex 7, France
[3] Laboratory of Organic Synthesis-Modeling and Optimization of Chemical Processes, Badji Mokhtar University, P.O. Box 12, 23000 Annaba, Algeria
[4] Ecole des Métiers de l'Environnement, Campus de Ker Lann, 35170 Bruz, France.
[5] Ecole Polytechnique Fédérale de Lausanne, EPFL-STI-LTP, Station 12, CH-1015 Lausanne, Switzerland.
* Corresponding authors: A.A. Assadi (aymen.assadi@ensc-rennes.fr) and S. Rtimi (sami.rtimi@epfl.ch)

Table S1. Second order rate constants of different scavengers with radical species

Scavengers	t-BuOH	i-PrOH	KI	BQ
Radicals species	k (M^{-1} s^{-1})	k (M^{-1} s^{-1})	k (M^{-1} s^{-1})	k (M^{-1} s^{-1})
•OH	6.0 x 10^8	1.9 x 10^9	1.1 x 10^{10}	1.35 x 10^9
$SO_4^{•-}$	8.0 x 10^5	1.9 x 10^9	-	-
h^+	-	-	1.1 x 10^{10}	-
$O_2^{•-}$	-	-	-	0.9-1.0 x 10^9
